# Expression and purification of the transmembrane domain of Fukutin-I for biophysical studies

**DOI:** 10.1016/j.pep.2010.01.019

**Published:** 2010-07

**Authors:** P. Marius, J.N. Wright, I.S. Findlow, P.T.F. Williamson

**Affiliations:** School of Biological Sciences, University of Southampton, Basset Crescent East, Southampton SO16 7PX, UK

**Keywords:** Expression, Purification, Transmembrane peptides, Solid-state NMR

## Abstract

Fukutin-I is a member of a family of putative O-linked glycosyltransferases linked to the glycosylation of the dystrophin complex. Mutations in this family of proteins have been linked to a number of congenital muscular dystrophies that arise from the hypoglycosylation of α-dystroglycan. Critical to the function of Fukutin and other members of this family is their localisation within the cell, which has been shown to depend critically on the interactions between the N-terminal transmembrane domain of these proteins and the lipid bilayer within the ER/Golgi. To investigate how the interactions between the N-terminal transmembrane domain and the lipid bilayer regulate the localisation of Fukutin-I, we have developed an efficient expression and purification protocol in *Escherichia coli* to allow biophysical studies to be performed. Expressing the N-terminal domain of Fukutin-1 fused to a His_6_ tag resulted in the localisation of the protein to the bacterial membrane. A purification strategy has been developed to isolate the highly hydrophobic transmembrane domain of Fukutin-1 from the membrane with yields of approximately 4 mg per litre of minimal media. Preliminary biophysical analyses have confirmed the identity of the peptide and revealed that in hydrophobic solvents mimicking the bilayer, the peptide adopts a well-structured α-helix as predicted from the sequence.

## Introduction

Recently, a number of genes have been identified that are involved in the O-linked glycosylation of α-dystroglycan (αDG)^1^, an important component of the dystrophin associated complex that anchors muscle fibres to the extracellular scaffold [1], these genes include *fukutin*, *fukutin related protein*, *LARGE*, *POMGnT1*, *POMT1* and *POMT2*. Mutations in these genes have been shown to result in the aberrant glycosylation of αDG resulting in a broad spectrum of congenital muscular dystrophies [1]. Sequence analysis suggests that these genes encode for Type-II membrane proteins with putative glycosyltransferase activity, an observation consistent with the hypoglycosylation of αDG detected in patients carrying mutations in any of these genes [1]. Subsequent studies have revealed that these proteins are normally located within the ER/Golgi complex in agreement with their proposed role in the glycosylation of αDG [2–5]. Interestingly, a number of the mutations associated with these genes result in the miss-localisation within the cell [3] suggesting that their retention within the ER/Golgi complex is vital for the appropriate glycosylation of αDG.

The retention of proteins, including glycosyltransferases, within the ER and Golgi is a highly dynamic process reliant on the tight regulation of both antero- and retrograde transport steps [6]. Sequence analysis and biochemical analyses have demonstrated that retrograde transport is largely regulated by the receptor mediated recognition of specific peptide motifs on the extra-membranous domains of ER and Golgi resident proteins [6]. In contrast, regulation of anterograde transport appears to be dependent on the shorter N-terminal transmembrane domain (TMD) that is typically found in ER and Golgi resident proteins [6]. It has been proposed that this interaction between the shortened N-terminal TMD and the atypical lipid composition found within the ER and Golgi complex plays an important role in regulating anterograde trafficking [7,8]. Indeed, in the context of muscular dystrophy, the N-terminal transmembrane domains of the proteins encoded by *fukutin* and *fukutin related protein* have been shown to be sufficient to retain the protein within the Golgi complex [9].

Although the role of the N-terminal TMD in the retention of these proteins is now known, a molecular understanding of this process remains to be elucidated. Several models based on lipid-mediated sorting and protein oligomerisation have been proposed [6,8,10,11]. To understand at a molecular level how lipids regulate protein trafficking, we are studying the transmembrane domain of the putative glycosyltransferase linked to Fukuyama muscular dystrophy encoded by the gene *fukutin*. In keeping with this family of proteins, the transmembrane domain of Fukutin has been demonstrated to be sufficient for the targeting of the protein to the Golgi apparatus [9]. Using a combination of liquid, solid-state NMR methods and other biophysical techniques, we are investigating how the differences in lipid bilayer composition affect the structure, oligomeric state and lateral segregation of Fukutin’s transmembrane domain.

A pre-requisite to these studies is the introduction of NMR sensitive isotopes into the protein of interest. Until recently, solid-state NMR studies of transmembrane peptides and proteins have relied on the introduction of labels site-selectively using solid-phase peptide synthesis. This technique permits the preparation of peptides up to 50 residues in length in milligram quantities sufficient for biophysical characterisation. However, recent advances in both liquid and solid-state NMR methodology rely on the uniform incorporation of NMR sensitive isotopes within the protein or peptides, a process that is costly when using solid-phase peptide synthesis, as uniformly labelled amino acids are required.

To exploit these advances in NMR methodology in the study of how lipids regulate Fukutin-1 trafficking, we have sought to develop an efficient bacterial expression system which permits both uniform and selective and extensive labelling of the transmembrane domain of Fukutin-1 thereby avoiding the synthesis of uniformly labelled peptide [12,13]. The expression and purification of transmembrane peptides and proteins is notoriously difficult due to their hydrophobicity and potential toxicity to the host cell [14]. To overcome these difficulties a number of groups have utilized carrier proteins to aid solubility, reduce toxicity and in some cases target the protein/peptide to the bacterial membranes [14]. However, it is our experience that after purification the yields obtained can be low as the desired peptides represent only a small fraction of the overall fusion protein and significant losses can be incurred during purification. Here we report on an efficient expression and purification method for the transmembrane domain of Fukutin-1 using simply a His_6_ tag for purification. Even in the absence of a classical bacterial membrane protein targeting sequence, the peptide is effectively targeted to the bacterial membrane presumably due to the intrinsic sequence and is expressed in quantities sufficient to support biophysical characterisation and introduction of isotopes for subsequent NMR studies.

## Materials and methods

Restriction enzymes *Sph1,* *Pst1 and Dpn1*, *Pfu* polymerase, T4 DNA ligase, thermosensitive alkaline phosphatase (TSAP) were purchased from Promega, UK. The pQE32 vector and M15 [PREP] *Escherichia coli* strain were bought from Qiagen, UK. The detergent, dodecylmaltoside (DDM) was supplied by Anatrace. Purification reagents were obtained from Sigma. ^15^NH_4_Cl and ^13^C-glucose were bought from Goss Scientific, UK. Oligonucleotides and sequencing analyses were obtained from Eurofins, MWG, UK.

### Construction of the expression plasmid

The protein sequence (MQRINKNVVL ALLTLTSSAF LLFQLYYYKH YLSARN) corresponding to the transmembrane domain of Fukutin-1 (UniProtKB ID: Q8R507) with associated flanking regions was reverse-translated with optimal codon usage for *E. coli* to generate a synthetic gene corresponding to the transmembrane domain of Fukutin-1. The oligonucleotide sequence was chemically synthesized and cloned into the pGS-21a vector (Genescript, New Jersey, USA). For protein expression, the gene was cloned into the pQE32 vector. The sequence encoding the FK1TMD was amplified by standard PCR at an annealing temperature of 61 °C using the forward primer GATATCGCATGCATGAGCCGTA and the reverse primer GTGGTGCTGCAGTTAGTTACGC [15]. The primers were designed to introduce the *Sph1* and *Pst1* restriction sites at the 5′ and 3′ end of the coding sequence, respectively. Following digestion of the PCR product with *SphI* and *PstI*, the purified PCR product was ligated into the pre-digested pQE32 vector at a molar ratio 3:1 resulting in the His_6_-FK1TMD plasmid, subsequently referred to as FK1TMD. The ligation mix was used to transform competent M15 [PREP4] *E. coli* cells. The sequence of the FK1TMD plasmid was subsequently verified by DNA sequencing.

### Overexpression of FK1TMD

An overnight culture (10 mL) of M15 *E. coli* transformed with the FK1TMD containing plasmid was grown on LB containing 100 μg/mL ampicillin and kanamycin 50 μg/mL at 37 °C. The overnight culture was used to inoculate 1 L LB medium supplemented with antibiotics and grown at 37 °C to an OD_600_ of 0.6. FK1TMD peptide overexpression was subsequently induced for 4 h at 37 °C by the addition of isopropyl-β-d-thiogalactopyranoside (IPTG) to a final concentration of 1 mM. For ^15^N and ^13^C-labelled FK1TMD, the overnight cultures were spun down and resuspended in 250 mL of M9 minimal medium containing 1 g/L ^15^NH_4_Cl and 3 g/L ^13^C-glucose, respectively instead of LB medium [15] and grown to OD_600_ of 0.6. The culture was then diluted to 1 L labelled minimal medium and grown to an OD_600_ of 0.6. Expression was induced by the addition of IPTG to a final concentration of 1 mM and grown for a further 4 h. Cells were harvested at 4 °C by centrifugation at 12000*g* for 20 min and pellet was stored at −20 °C.

### Purification of FK1TMD

The cell pellet was resuspended and lysed in 40 mL phosphate buffered saline (PBS) containing 50 mM imidazole and 1 mM phenylmethylsulphonyl fluoride (PMSF), pH 7.5 and sonicated on ice for 5 min: 15 s on; 20 s off at power level 7 (Misonix sonicator). The lysate was clarified by ultracentrifugation at 142,000*g* for 35 min. The pellet was resuspended in 50 mL solubilisation buffer (PBS containing 20 mM DDM, 50 mM imidazole, pH 7.5) for 1 h at room temperature. The solubilised fraction was clarified by centrifugation at 21,000*g* and the supernatant loaded onto a Ni^2+^-NTA affinity column (GE Healthcare) pre-equilibrated with lysis buffer. The column was washed with 15 bed volumes of washing buffer (PBS, 200 mM imidazole, 1 mM DDM, pH 7.5) and the peptide eluted in PBS containing 600 mM imidazole and 1 mM DDM, pH 7.5. The eluted fractions were analysed by SDS–PAGE using a 20% tricine gel at 125 V for 90 min [16]. Gels were stained with Sypro-Orange (Invitrogen) in 10% acetic acid for 40 min. After destaining in 10% acetic acid for 2 min, the gel was visualised using a UV transilluminator. For Western Blot analysis the destained proteins were transferred to a polyvinylidene fluoride (PVDF) membrane using a voltage of 70 V for 25 min. The fusion protein was detected using an antibody against polyhistidine (Sigma) at a dilution of 1:4000. To precipitate the protein out of solution, it was dialyzed against distilled deionized water using a 2 kDa molecular weight cut-off Slide-a-lyzer dialysis cassette (GE Healthcare). Three buffer exchanges (2 h each) were performed to remove imidazole and to dilute DDM to a concentration below its CMC (170 μM). The pellet was collected after centrifugation at 229,000*g* for 30 min and washed with distilled water. The peptide was then dissolved in acetonitrile:water (50:50) containing 0.1% trifluoroacetic acid (TFA) and lyophilized to white powder.

### Circular dichroism

The CD spectrum of FK1TMD peptide dissolved in trifluoroethanol (Sigma) to a final concentration of 0.15 mg/mL was measured using a Jasco J720 spectropolarimeter and a 1 mm-path-length quartz cuvette (Hellma) at room temperature. The spectrum scan was performed from 300 to 190 nm using a spectral bandwidth of 2 nm, scanning speed of 100 nm min^−1^ and 4 s response time. Data was analysed using the CONTILL analysis algorithm on the DichroWeb server [17] using the reference dataset 4.

### Mass spectrometry

The purity of the peptide FK1TMD was confirmed by mass spectrometry. Mass spectra were recorded on an LCTTM (Waters, UK) orthogonal acceleration time-of-flight mass spectrometer fitted with a nano electospray source. FK1TMD in TFE (5 pmol/μl, 5 μl) was loaded into borosilicate capillaries (1.2 mm o.d. × 0.69 mm i.d.) (Clark Electromedical Instruments, Reading, UK) that had been drawn to a fine tip using a micro-electrode puller (Narishige, Tokyo, Japan) and sputter coated with gold/palladium. Spectra were recorded in the positive ion mode between 600–1500 *m/z* using the following instrument settings: capillary 1200 V, sample cone 35 V, extraction cone 9 V, source temperature 50 °C. Typically, 100 spectra were combined and deconvoluted using the maximum entropy algorithm MaxEntTM (Micromass, Altrincham, UK) to give relative molecular mass spectra over the range 5000–7000 Da at 1 Da resolution. Spectra were externally calibrated using NaI/CsI spectra recorded under identical conditions immediately after each sample.

### Liquid-state NMR

Liquid-state NMR spectra were acquired on a Varian Inova 600 MHz spectrometer equipped with a triple resonance probe equipped with gradients at 35 °C. The sample was prepared by dissolving 4 mg of the lyophilized FK1TMD in 0.5 mL d_2_-trifluoroethanol. ^1^H/^15^N HSQC spectra were acquired with states phase sensitive detection in the indirect dimension. Spectra were acquired with 40 (*t*_1_, ^15^N) by 639 (*t*_2_, ^1^H) complex data points in each dimension with spectral widths of 2 and 10 kHz, respectively. The data was processed using NMRPipe [18]. Indirect dimension data points were linear predicted to 128 data points and a sine-bell squared filter applied in both the direct and indirect dimensions. The resulting data was zero-filled before Fourier transformation to give a data matrix of 512 by 4096.

## Results and discussion

### Construct design and expression

**Fig. 1:**
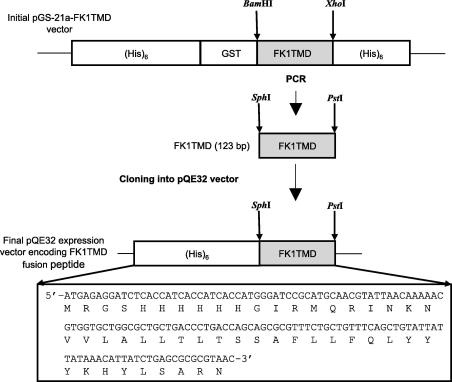
Schematic representation of the expression cassette for the His-tagged fusion peptides FK1TMD.

**Fig. 2:**
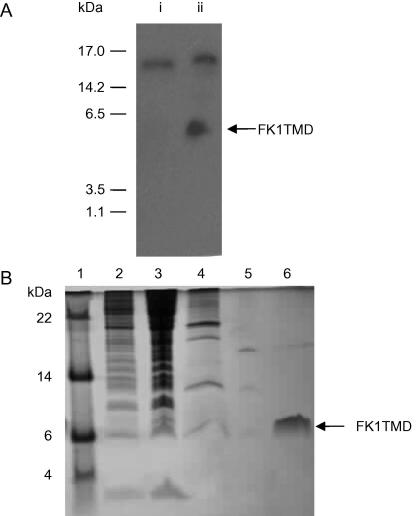
Analysis of the expression and purification of FK1TMD. (A) Western blot analysis showing the induction of FK1TMD following the addition of IPTG. 1 ml aliquot of pre- (i) and post- (ii) induction cell lysate was probed with anti-His_6_ antibody (1:4000). (B) Tricine gel (20%) of a typical purification of FK1TMD using Ni-NTA affinity column stained with the fluorescent SYPRO-Orange. Lane 1, molecular weight markers; Lanes 2 and 3, uninduced (5 μL) and induced bacterial cell lysate (15 μL); Lane 4, solubilised membrane fraction (5 μL); Lane 5, final wash prior to elution (5 μL) and Lane 6, purified FK1TMD (15 μg) as fusion peptide.

The expression cassette for the expression of FK1TMD and an overview of the cloning strategy are given in Fig. 1. DNA sequencing results confirmed the insertion of the FK1TMD gene into the pQE32 vector and the absence of point mutations. After transformation of M15 [PREP4] competent cells with the FK1TMD expression plasmid, the optimal expression conditions for FK1TMD in LB medium were determined by monitoring bacterial growth and expression time courses under varying temperature and IPTG concentrations and analyzing the resulting cell lysates by Western Blot using an antibody against the His_6_ tag (data not shown). Optimal peptide expression was observed following induction for 4 h with 1 mM IPTG as shown by Western blot using an antibody that recognises the His_6_ tag (Fig. 2A). The FK1TMD ran as a single monomeric band of approximately 6.0 kDa. An additional high molecular weight endogenous bacterial protein is also recognised by the antibody against the His_6_ tag. For experiments which required uniformly labelled with ^13^C-carbon and ^15^N-nitrogen, overnight culture was grown in LB medium, spun down and grown up to 1 L in minimal medium in two stages as described above.

### Purification of FK1TMD

Preliminary analysis with cell lysate revealed that FK1TMD was mainly localised in the insoluble cellular fraction (data not shown). The extraction of the FK1TMD peptide required solubilisation of the membrane fraction with the non-ionic detergent, DDM, for 1 h. The FK1TMD was subsequent purified by Ni-NTA affinity chromatography. The yield of the pure peptide eluted from the Ni^2+^-NTA affinity column was 4 ± 2 mg/L as determined by a standard BCA assay (Bio-rad) (Table 1). We note that extraction with alternative detergents, in particular Triton ×100, resulted in higher yields of purified protein. However, these detergents proved difficult to remove hindering subsequent analysis by electrospray mass spectroscopy. The purified FK1TMD was subsequently precipitated out of solution by dialysis against deionised water until the DDM concentration was below its CMC of 170 μM. After pelleting of the insoluble material and washing with deionized water, the resulting peptide was dissolved in 50:50 acetonitrile:water with 0.05% TFA and lyophilised to a white fluffy powder. In this form, the pellet was ready for further characterisation.

The purity of the peptide was verified on a 20% SDS–PAGE tricine gel using Sypro-Orange fluorescent stain with the dominant band from FK1TMD running slightly higher than expected at approximately 6.0 kDa (Fig. 2B). The endogenous *E. coli* protein identified by Western blot analysis also bound to the Ni^2+^-NTA affinity column and was removed by a high stringency wash with 200 mM imidazole prior to elution of the FK1TMD resulting in a final purity in excess of 95% as determined from the gel. It is noted that Sypro-Orange staining offered improved detection over Commassie for hydrophobic peptides presumably reflecting the more hydrophilic nature of the solvents used which prevents the dissolution of the hydrophobic peptide from the gel. The enhanced detection with Sypro-Orange proved invaluable for the efficient detection of the FK1TMD.

FK1TMD labelled with ^15^N and/or ^13^C- was prepared from *E. coli* harvested from minimal media as described in the material and methods. Following purification the yield was approximately 20% less that that obtained from similar growths on LB media.

### Mass spectroscopic analysis of FK1TMD

**Fig. 3:**
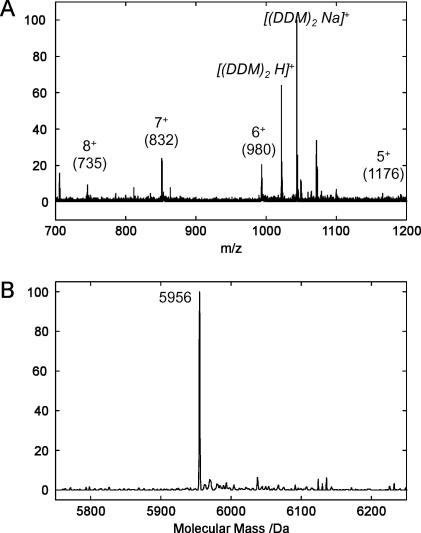
Mass spectrum of the FK1TMD. (A) Spectrum showing the mass to charge ratios of various species after electrospray mass spectrometry. The labelled peaks indicate the multiply charged states of FK1TMD. Peaks correlating to the dimers of the detergent, DDM (*M*_W_ of 510 Da), associated with the peptide were also dominant (shown in italics). Data has been normalised to the value obtained for the largest peak. (B) The deconvoluted spectrum shows a single peak at 5956 Da corresponding to the molecular weight of the ^15^N-labelled FK1TMD.

The sequence of the FK1TMD peptide including His-tag and linker region is MRGSHHHHHH GIRMQRINKN VVLALLTLTS SAFLLFQLYY YKHYLSARN with a predicted molecular mass of 5,874 Da (Expasy Protparam, [19]). Electrospray mass spectra of the ^15^N labelled FK1TMD purified from DDM always contained a significant contribution from the detergent with peaks corresponding to the H^+^ and Na^+^ salts present (Fig. 3A). In addition, peaks arising from the peptide are observed at *m*/*z* ratios of 1176, 980, 832 and 735 corresponding to the 5^+^, 6^+^, 7^+^ and 8^+^ charged states respectively (Fig. 3A), with MaxEnt analysis (Fig. 3B) calculating a peptide mass of 5,956 Da. This is in perfect agreement with the predicted molecular weight of the peptide given that each of the 82 nitrogens within the peptide is enriched at 99% with nitrogen-15.

### Circular dichroism

**Fig. 4:**
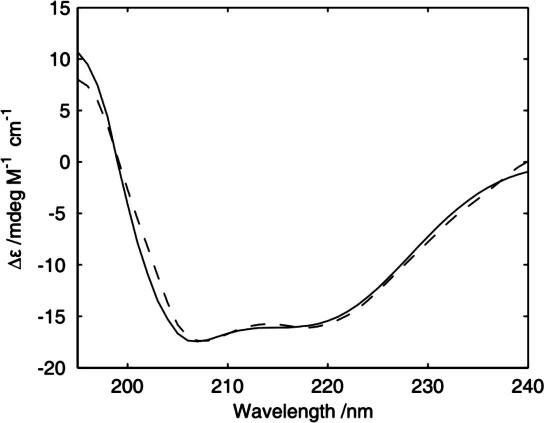
CD spectrum of FK1TMD in trifluoroethanol (0.15 mg ml^−1^) revealing the expected α-helical conformation for the TMD of FK1 in TFE (solid line, experimental; dashed, fitted).

Circular dichroism was used to determine the secondary structure of FK1TMD peptide in TFE. The CD spectrum possessed two minima at 208 and 222 nm consistent with the peptide adopting an α-helical conformation (Fig. 4). Analysis with the Contill algorithm [17] using basis set 4 indicated that the alpha helical content of FK1TMD was in the region of 85 ± 15% in TFE with the remainder composed of disordered, loop and strand structures. This is in good agreement with the hydropathy analysis (TMPred, Expasy) which suggests that residues Asn_7_–Leu_32_ or 75% of the FK1TMD peptide should adopt an α-helical conformation in TFE.

### Solution-state NMR

**Fig. 5:**
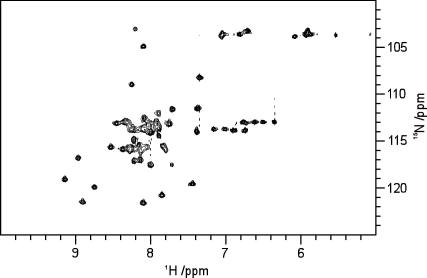
^1^H/^15^N-HSQC resonances of FK1TMD in d_2_-trifluoroethanol at 25 °C showing over 90% of the peaks expected from the 49 residue FK1TMD.

^1^H/^15^N-heteronuclear single-quantum coherence (HSQC) liquid-state NMR spectra [20] were acquired to characterise and confirm the purity of FK1TMD. The spectrum of the lyophilized FK1TMD peptide dissolved in d_2_-TFE (4 mg/mL) (Fig. 5) showed good resolution and peak dispersion. Of the 49 peaks expected that corresponding to the backbone amide groups 46 were apparent and resolved. In addition a number of resonances are clearly visible that arise from nitrogens in the amino acid sidechains. The broad distribution of resonances within the HSQC spectrum indicates, as expected, that in TFE the FK1TMD is structured in agreement with the CD spectra.

## Conclusion

In this work we have described an efficient method for production of the transmembrane domain of the putative glycosyltransferase Fukutin-1, yielding milligram quantities of peptide on minimal media making the systems suitable for the production of the FK1TMD for biophysical analysis including the introduction of isotopes for NMR experimentation. In contrast to earlier published methods that rely on the expression of the transmembrane domains fused to large carrier proteins, improving solubility and aiding in both the expression and purification, here just a single His_6_ tag has been added to the transmembrane domain to aid purification with the transmembrane domain of Fukutin-1, apparently sufficient to target the protein to the bacterial membrane. Although the overall mass of recombinant protein is less than that obtained from systems employing larger fusion proteins [14] a greater percentage mass of the recombinant protein expressed is the peptide of interest resulting in comparable yields for the final purified peptide. Furthermore, the smaller affinity tag employed suggests that, in favourable cases where the His_6_ tag does not interfere unduly with the physical properties of the peptide being studied, the peptide can be used without cleavage from the affinity tag and subsequent purification. We acknowledge that the expression of the transmembrane domain of integral membrane proteins will depend greatly on the particular sequence being studied, however the protocol proposed here offers an alternative route to the efficient and economic expression and labelling of such domains for NMR and other biophysical studies. The protocols developed will allow the investigation into the role interactions between the N-terminal transmembrane of Fukutin-I and the lipid bilayer plays in its retention in the ER/Golgi.

## Figures and Tables

**Table 1:** Purification of FK1TMD. Figures given are for a 1 L growth on LB and are representative of three separate trials.

Fraction	Protein content (mg)	Purity (%)
Mass of wet cell pellet	5200	
Cell lysate	560	
Solubilized membrane fraction	148	
FK1TMD after Ni-affinity chromatography	4.0	95

## Footnotes

1 *Abbreviations used*: αDG, α-dystroglycan; TSAP, thermosensitive alkaline phosphatase; DDM, dodecylmaltoside; IPTG, isopropyl-β-d-thiogalactopyranoside; PBS, phosphate buffered saline; PMSF, phenylmethylsulphonyl fluoride; PVDF, polyvinylidene fluoride; TFA, trifluoroacetic acid; HSQC, ^1^H/^15^N-heteronuclear single-quantum coherence.
